# The interaction between paracetamol and ethanol during the early development of zebrafish

**DOI:** 10.3389/fphar.2026.1858736

**Published:** 2026-08-25

**Authors:** Anna Małkowska, Karolina Wieczorek, Kacper Pawlak, Julia Elżbieta Bonder

**Affiliations:** 1 Department of Toxicology and Food Science, Faculty of Pharmacy, Medical University of Warsaw, Warsaw, Poland; 2 Zebratox Student Research Group, Faculty of Pharmacy, Medical University of Warsaw, Warsaw, Poland

**Keywords:** ethanol, FASD, interaction, paracetamol, zebrafish

## Abstract

**Introduction:**

The issue of drug-alcohol interactions is a major concern in drug therapy and can endanger patient safety. Paracetamol is one of the most widely used analgetic drugs, while ethanol is commonly consumed addictive substance. This study investigated the toxic effects of paracetamol, ethanol, and their mixture at different concentrations on the embryonic development of zebrafish (Danio rerio, D. rerio).

**Methods:**

Zebrafish embryos (20 embryos per group, 3 biological replicates for each concentration or combination of concentrations) were exposed for 72 h post-fertilization to paracetamol (0.5, 1.0, 2.0, 3.0, 5.0, and 10.0 mM), ethanol (85.5, 171.0, 256.5, 342.0, 513.0, 855.0) or their combinations. Additionally, combinations of paracetamol and ethanol were examined to assess the type of interaction (0.5:85.5; 1.0:171.0; 1.5:256.5; 2.0:342.0; 3.0:513.0; and 5.0: 855.0 mM).

**Results:**

The combination of the drug and alcohol resulted in a higher incidence of developmental defects compared to the effects of either substance used alone. The most frequently observed changes included decreased larval pigmentation, pericardial and sac edema or increased larval mortality compared to control (e.g., 2-4 fold increase over control). Notably, there was a statistically significant reduction in both eye size and body length of the larvae. Furthermore, the severity of the defects increased proportionally with dose. Additionally, analysis using the Chou-Talalay method revealed the synergism in this interaction, with a combination index below 1.

**Discussion:**

These findings demonstrate that exposure to paracetamol and ethanol exerts a synergistic toxic effect on early zebrafish development, emphasising the potential risks associated with drug-alcohol co-exposure.

## Introduction

An important area of research in pharmacology is the study of interactions between drugs, and drug-alcohol (Chan and Anderson, 2014; Jang and Harris, 2007).

Paracetamol (acetaminophen) is a widely used over-the-counter (OTC) medication for treating mild pain and is often used as an adjunct in managing severe pain (the first step of the analgesic ladder) and fever (Freo et al., 2021). Although this active metabolite of phenacetin has been in use since 1887, its mechanism of action is still not fully understood. Its analgesic effect is likely due to the inhibition of cyclooxygenase (COX) in the central nervous system (CNS) (Bertolini et al., 2006). When taken in recommended doses, paracetamol is considered to have a high safety profile, including for use by pregnant women (de Castro et al., 2022).

However, recent studies have shown that children whose mothers took paracetamol during pregnancy may experience neurological and behavioural issues, and paracetamol should be taken with caution (Bauer et al., 2021; Prada et al., 2025; Sznajder et al., 2022; Woodbury et al., 2024).

Paracetamol is primarily metabolized in the liver via glucuronidation and sulfation. A small amount is oxidized by cytochrome P450 enzymes, mainly CYP2E1, to form *N*-acetyl-p-benzoquinone imine (NAPQI), a highly reactive, hepatotoxic metabolite. Under physiological conditions, NAPQI is usually detoxified by glutathione conjugation. Ethanol modifies this pathway by inducing enzymes and depleting glutathione, altering hepatotoxic risk. Ethanol can induce enzymes (like CYP2E1), potentially raising the risk of liver damage (Prescott, 2000; Tanaka et al., 2000). The nature of the interaction depends on the pattern of ethanol consumption, with chronic exposure leading to CYP2E1 induction and an increased risk of paracetamol toxicity, while acute exposure to ethanol can transiently inhibit this metabolic pathway (Ghosh et al., 2021; Mian et al., 2020).

Both paracetamol and ethanol can induce oxidative stress, and simultaneous exposure to both may exacerbate this effect, resulting in the accumulation of reactive oxygen species (ROS), mitochondrial damage and oxidative stress (Liao et al., 2023).

Ethanol is the most widely used psychoactive substance and is present in a variety of alcoholic beverages. However, it poses a significant public health threat due to its high potential for abuse and its considerable impact on human health. Ethanol is often consumed with other drugs, leading to potential interactions (Chan and Anderson, 2014). The effects of alcohol on the body are complex, influencing various enzymatic and neurochemical pathways (Pervin and Stephen, 2021). Acetaldehyde, the primary metabolite of ethanol, is responsible for mutagenic and carcinogenic effects. Additionally, ethanol acts as a co-carcinogen, increasing the risk of cancer development when it interacts with other carcinogens in the body (Seitz and Stickel, 2010).

Prenatal exposure to ethanol can lead to the development of Fetal Alcohol Syndrome (FAS) and Fetal Alcohol Spectrum Disorder (FASD). These conditions result in significant abnormalities in a child’s physical, motor, sensory, and emotional development (Muralidharan et al., 2015). Children affected by these disorders may experience a range of mental and physical challenges (Popova et al., 2023; Sokol et al., 2003; Williams et al., 2015).

Previous studies indicate the scientific literature indicates that both paracetamol and ethanol can cause various side effects, including liver damage (Ghosh et al., 2021; Liu et al., 2021). Consequently, simultaneous consumption of these substances may potentiate their toxic effects (Gonçalves et al., 2024). An interaction related to a common metabolic pathway can occur when paracetamol is used by individuals who consume or abuse ethanol (Prescott, 2000).

Paracetamol is one of the most commonly used drugs during pregnancy and readily crosses the placental barrier, leading to fetal exposure to concentrations similar to those observed in maternal circulation. Available data also indicates that fetal detoxification capabilities, the activity of enzymes responsible for biotransformation of reactive metabolites and glutathione levels are limited. Additionally, the metabolism of paracetamol in the zebrafish model shows similarity to the pharmacokinetics observed in humans (Kantae et al., 2016; Patton et al., 2021). This suggests that the embryo may be particularly susceptible to synergistic or additive effects resulting from simultaneous exposure to paracetamol and other substances (Huang et al., 2025; Mian et al., 2020).

Despite well-characterised interactions between paracetamol and ethanol in adults and numerous studies examining their separate effects on prenatal development, no studies have examined their combined effects during early embryonic development (Chen et al., 2024; Karimi et al., 2015; Lu et al., 2023). This gap has significant biological and clinical implications, particularly in the context of the widespread use of paracetamol during pregnancy and the possibility of inadvertent exposure to ethanol in very early, often unrecognized stages of pregnancy (Prada et al., 2025). Research interaction during embryonic development also appears significant due to differences in enzyme activity—including, amongst others, cytochrome P450—between the developing fetus and adults (Robinson et al., 2020).

An example of a model that can aid in assessing interactions is the zebrafish. *D. rerio,* a member of the carp family, is a well-established model organism for *in vivo* toxicological studies (Cassar et al., 2020; Lima et al., 2026; Verma et al., 2024; Zhao et al., 2024). Zebrafish *(D*. *rerio)* are currently considered one of the most promising vertebrate models for studying pharmacological and toxicological interactions because they combine the biological complexity of vertebrates with high experimental throughput (Wang et al., 2025). Unlike mammalian models, zebrafish allow testing a large number of substances and dose combinations in a short time, which is crucial for analysing drug-drug and drug-alcohol interactions, where multiple co-exposure variants must be assessed (Tanguay, 2025).

A particular advantage of zebrafish is their high sensitivity to additive and synergistic effects (Csenki et al., 2021; Gomes et al., 2024; Hinfray et al., 2016; Liu et al., 2025; Min et al., 2025). Pharmacological interactions often become apparent only when several substances are simultaneously exposed, rather than in response to a single exposure. Zebrafish embryos and larvae exhibit high sensitivity to subtle developmental and functional changes (Jakobs et al., 2020; Nishimura et al., 2016).

The zebrafish model also enables simultaneous assessment of the effects of interactions at the developmental and functional levels of the nervous system. This is particularly important in the context of drug-alcohol interactions, as alcohol can exacerbate their impact on developmental processes. Zebrafish allows for parallel analysis of morphological and behavioral parameters, such as locomotor activity and responses to stimuli, enabling a more comprehensive assessment of the consequences of co-exposure (Bilotta et al., 2004; Horzmann and Freeman, 2018).

This popularity stems from several advantages it offers. One significant benefit is that its transparent embryos develop outside the body, allowing researchers to observe the entire development process from fertilization (Gamse and Gorelick, 2016). The complete development cycle takes approximately 3 months, and by just 48 h post-fertilization (hpf), the embryos have already formed basic organ systems, including the heart and blood vessels. Fish farming with zebrafish is relatively easy and cost-effective. Additionally, this small freshwater fish has fully sequenced genetic material that can be easily manipulated (Teame et al., 2019). Howe et al. (2014) indicates that zebrafish and humans share a genetic similarity of 70%–80% (Howe et al., 2014). As a result, zebrafish are used in a wide range of research areas, including toxicology, environmental studies, pharmacology, genetics, metabolic research, cancer studies, and more (Bambino and Chu, 2017; Adhish and Manjubala, 2023; Patton et al., 2021). The *D. rerio* embryo model is used to investigate toxicological effects and mechanisms (Zhao et al., 2024) and can help study interactions in toxicology research (Bustad et al., 2024).

The ethanol-induced developmental anomalies make *D*. *rerio* an excellent model for studying the prenatal teratogenic effects of alcohol (Manikandan et al., 2022; Zhang et al., 2014). The morphogenetic changes observed in *D*. *rerio* are akin to those seen in children with FASD, indicating that the underlying mechanisms may share commonalities. Nonetheless, this model is still in the ongoing model optimization phase, which could enhance our understanding of the mechanisms through which toxic substances affect a developing organism (Ben Lovely and Eberhart, 2014; Ben Lovely et al., 2016; Bilotta et al., 2004; Pinheiro-da-Silva & Luchiari, 2021).

The Chou-Talalay method is used to assess interactions between xenobiotics (synergistic, additive, or antagonistic) (Chou, 2010).

The use of this method in the zebrafish model allows for the generation of quantitative, dose-dependent endpoints and full dose-effect curves for single substances and their combinations (Lima et al., 2026). Many standard endpoints used in zebrafish toxicology (e.g., mortality, incidence of developmental abnormalities, measurable phenotypic changes) are graded and can be expressed as fraction affected (Fa), which satisfies the basic mathematical assumptions of the Chou–Talalay method. Evaluating the characteristics of synergism and additive interactions has already been the subject of previous work in zebrafish (Chang et al., 2020; Fan et al., 2021).

This study aimed to investigate the embryotoxic interactions between paracetamol and ethanol in an experimental model using *D. rerio*. To gather information about the potential effects of this interaction on the developing organism, we conducted a study utilizing an embryonic model of *D*. *rerio*. Embryos were exposed to varying doses of both paracetamol and ethanol, and the effects on morphogenetic and physiological changes were measured.

## Materials and methods

### The zebrafish experiments

Zebrafish embryos ABxTL were obtained from the International Institute of Molecular and Cell Biology in Warsaw (IMaCB) and maintained in E3 medium. All animals were maintained in the Zebrafish Core Facility, a licensed breeding and research facility (PL14656251, registry of the District Veterinary Inspectorate in Warsaw; 064 and 051, registry of the Ministry of Science and Higher Education) at the International Institute of Molecular and Cell Biology in Warsaw. Adult zebrafish and larvae were kept in E3 medium (2.48 mM NaCl, 0.09 mM KCl, 0.164 mM CaCl_2_·2H_2_O, and 0.428 mM MgCl_2_·6H_2_O) at 28.5 °C. The embryos were identified with staged according to (Kimmel et al., 1995) and OECD TG 236 (OECD, 2013). Only fertilized embryos gastrula stage (6 h post-fertilization (hpf)), were selected for the study. The selected embryos were placed in sterile, 96-well plates (3 biological replicates, 20 embryos per one concentration or tested mixture) containing the prepared solutions and control groups.

The concentrations of paracetamol were 0.5, 1.0, 2.0, 3.0, 5.0, and 10.0 mM, as reported in previous publications (Cedron et al., 2020; de Carvalho et al., 2024).

The ethanol solutions were 85.5, 171.0, 256.5, 342.0, 513.0, 855.0 mM as reported in earlier publications (Pinheiro-da-Silva & Luchiari, 2021; Ramlan et al., 2017).

The paracetamol was dissolved in E3 or in corresponding ethanol concentrations to obtain the examined solution of the mixture. The total amount of the solution was 200 µL per well. The solutions were not changed during this experiment.

The following mixtures of ethanol alone at concentrations of 85.5, 171.0, 256.5 and 342 mM and paracetamol in combination at 0.5, 1.0 and 2.0 mM were prepared to evaluate the effect of the mixture on mortality and the number of sublethal lesions (pericardial and yolk oedema, morphological measurements like length, width of the larvae, eye size and changes in pigmentation).

To assess the type of interaction using the Chou-Talalay model, solutions of paracetamol and ethanol mixtures with a constant concentration ratio 171 were prepared. Finally, mixtures containing 0.5:85.5; 1.0:171.0; 1.5:256.5; 2:342.0; 3:513.0 and 5:855.0 mM (paracetamol: ethanol) were obtained.

Observations were conducted at 24-h intervals up to 72 h post-fertilization (hpf). The plates were incubated at a constant temperature of 28.5 °C, with a light-dark cycle of 12 h each throughout the study period. All experiments were repeated three times.

The embryos were examined under a microscope (Olympus CKX53) equipped with an Olympus 2x/0.06 lens, and images were captured using an Olympus EP50 camera (CAM-EP50, Olympus, Tokyo, Japan). The length, width, and pericardial edema area of the control and experimental group embryos, which had emerged from their chorions, were measured using the EPview version 1.3 software. A P0-P2 scale, developed in-house was used to assess pigmentation.

After completing the experiment, zebrafish larvae were euthanized by immersion in tricaine solution. Larvae were exposed to a buffered tricaine solution at a concentration of 300 mg/L (pH adjusted to 7.0–7.5 with sodium bicarbonate).

All procedures were conducted in accordance with the European Directive 2010/63/EU on the protection of animals used for scientific purposes and complied with relevant national (Polish) regulations.

CompuSyn software (ComboSyn, Inc. NJ, USA) is utilized to analyze the interactions between chemical compounds, particularly *in vitro* and *in vivo* studies involving combinations of anticancer drugs. The software operates based on the Chou-Talalay method, which employs the median effect equation derived from the law of mass action. This method utilizes algorithms based on the law of mass action to determine the Dm-value, which signifies the median effective dose, and the m-value, which describes the shape of the dose-effect curve. The median effect equation underpins the combination index (Cl) equation, enabling quantification of interactions between xenobiotics. When Cl is greater than 1, it signals an antagonistic effect; when Cl equals 1, it indicates an additive effect; and when Cl is less than 1, it signifies a synergistic effect (Chou, 2006). Using the CompuSyn program and the Chou-Talalay method, we determined the LC_50_ values for paracetamol, ethanol, and their mixture and the Cl (combination index).

### Chemicals and reagents

Paracetamol (CAS number 103–90–2) and ethyl 3-aminobenzoate methane sulfonate (Tricaine, 98% pure, CAS number 886–86–2) were purchased from Sigma-Aldrich (St Louis, MO, USA). Absolute ethyl alcohol (99.8% pure, CAS number 64–17–5) was obtained from POCH (Gliwice, Poland). Paracetamol was dissolved in either E3 medium or absolute ethyl alcohol. NaCl (CAS number 7647–14–5), KCl (CAS number 7447–40–7), CaCl_2_·2H_2_O (CAS number 10043–52–4) and MgCl_2_·6H_2_O (CAS number 7791–18–6) were purchased from Chempur (Piekary Śląskie, Poland).

### Statistical analysis

Statistically significant differences between groups were evaluated using one way ANOVA followed by the HSD Tukey’s test, when the groups show normality (Shapiro-Wilk’s test) and homogeneity (Leven’s test) or the non-parametric Kruskal–Wallis test with Dunn’s *post hoc* tests, with Bonferroni correction. Results were considered significant at *p*-value <0.05. Analyses were conducted using Statistica 13.3 software (StatSoft, Poland) and GrahPad Prism 10.6.1 (GraphPad Software, USA).

## Results

### Summary of lethal and sublethal changes

Lethal changes were assessed by observing coagulation of fertilized eggs, lack of somite formation, lack of detachment of tail from the yolk and the absence of heartbeat (OECD, 2013). The assessment of sublethal changes at 72 hpf included pericardial edema, yolk edema, tail edema, tail curvature, scoliosis, lordosis, general deformities, and failure to hatch.

Paracetamol administered alone at concentrations of 0.5, 1.0, and 2.0 mM did not affect mortality or induce sublethal changes such as pericardial or yolk sac edema. At these concentrations, paracetamol did not cause any statistically significant alterations in the evaluated endpoints (data not shown).

Ethanol alone in concentrations of 171.0 mM and 342.0 mM may cause changes in mortality, the occurrence of cardiac and yolk diseases, the effect of which was presented in previous works (Fu et al., 2021; Malkowska et al., 2024; Pinheiro-da-Silva and Luchiari, 2021; Ramlan et al., 2017).

**FIGURE 1:**
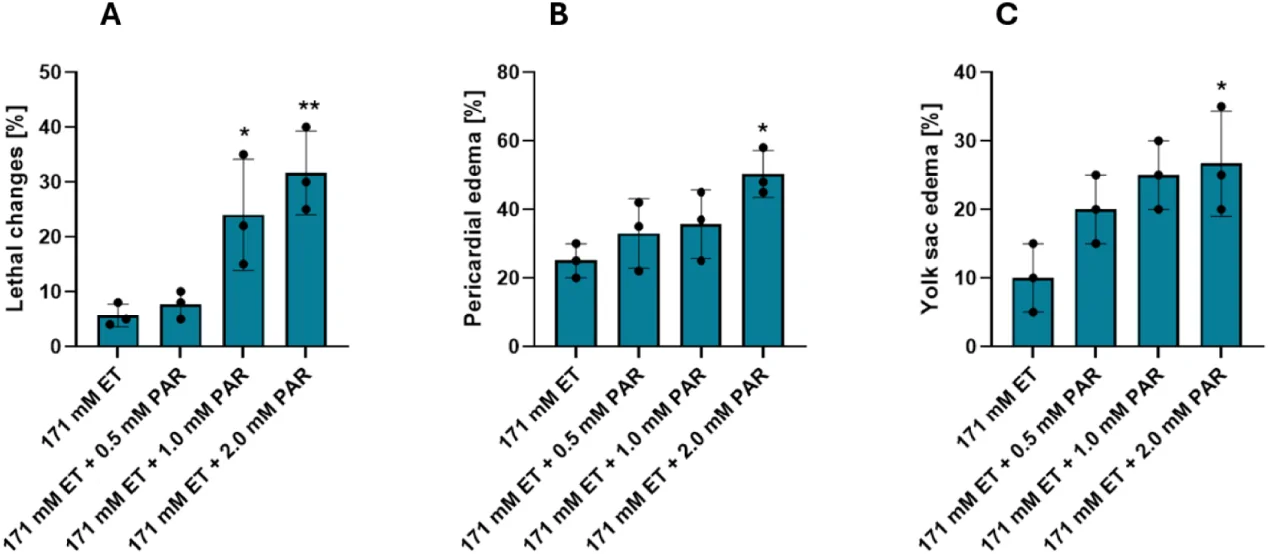
Lethal **(A)** and sublethal effects **(B)** pericardial edema and **(C)** yolk sac edema observed in zebrafish embryos at 72 hpf following continuous exposure to ethanol at 171.0 mM (ET), which served as the control group, and in groups co-exposed to paracetamol (PAR; 0.5, 1.0, and 2.0 mM) in combination with 171.0 mM ethanol. Results are presented as mean ± standard deviation (SD) from three biological replicates, with 20 embryos per group. Statistical analysis was performed using one-way analysis of variance (ANOVA) followed by Tukey’s HSD *post hoc* test. *p < 0.05, **p < 0.01 vs. the control group (171.0 mM ethanol). ET–ethanol; PAR–paracetamol.

Exposure to 171.0 mM (1%) ethanol and paracetamol at 0.5, 1.0, and 2.0 mM, used simultaneously, significantly potentiated the lethal effect and sublethal changes, such as pericardial and yolk sac edema, compared with 171.0 mM ethanol alone (Figure 1).

**FIGURE 2:**
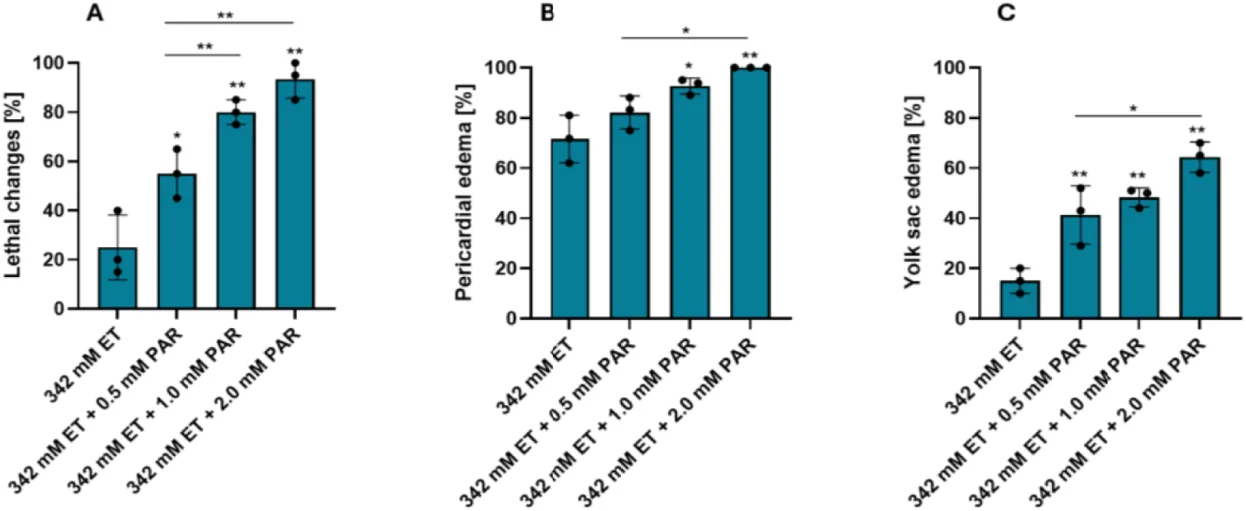
Lethal **(A)** and sublethal effects **(B)** pericardial edema and **(C)** yolk sac edema observed in zebrafish embryos at 72 hpf following continuous exposure to 342.0 mM ethanol (ET), as well as in groups exposed to paracetamol (PAR; 0.5, 1.0, and 2.0 mM) in combination with 342.0 mM ethanol. Ethanol at 342 mM served as the control group. Results are presented as mean ± standard deviation (SD) from three biological replicates, with 20 embryos per group. Statistical analysis was performed using one-way analysis of variance (ANOVA) followed by Tukey’s HSD *post hoc* test. *p < 0.05, **p < 0.01 vs. 342.0 mM ethanol group. ET–ethanol; PAR–paracetamol.

A similar effect was observed when exposed to 342.0 mM (2%) ethanol and paracetamol at 0.5, 1.0, and 2.0 mM, used simultaneously, which significantly potentiated the lethal effect and sublethal changes, such as pericardial and yolk sac edema, compared with 342.0 mM ethanol alone (Figure 2).

Exposure to 171.0 mM ethanol combined with 2 mM paracetamol resulted in greater effects (lethal changes, pericardial edema and yolk sac edema), whereas with 342.0 mM ethanol, differences in mortality and the number of edemas were already evident with 0.5 mM paracetamol administered concomitantly with 342.0 mM ethanol. Additionally, statistically significant differences were observed between 342.0 mM ethanol with 0.5 mM paracetamol and 342.0 mM ethanol with 1.0 mM paracetamol, and between 342 mM ethanol with 0.5 mM paracetamol and 342.0 mM ethanol with 2.0 mM paracetamol. Exposure to the combination of paracetamol (0.5, 1.0 and 2.0 mM) with 85.5 mM ethanol, did not produce greater changes, such as edema or mortality, than the 85.5 mM ethanol alone.

In summary, the collected data indicated that increased mortality and sublethal changes occurred with rising concentrations of the tested compounds.

Additionally, the combination of paracetamol and ethanol led to a higher incidence of defects than separate exposure to either substance alone.

### Assessment of edema occurrence

**FIGURE 3:**
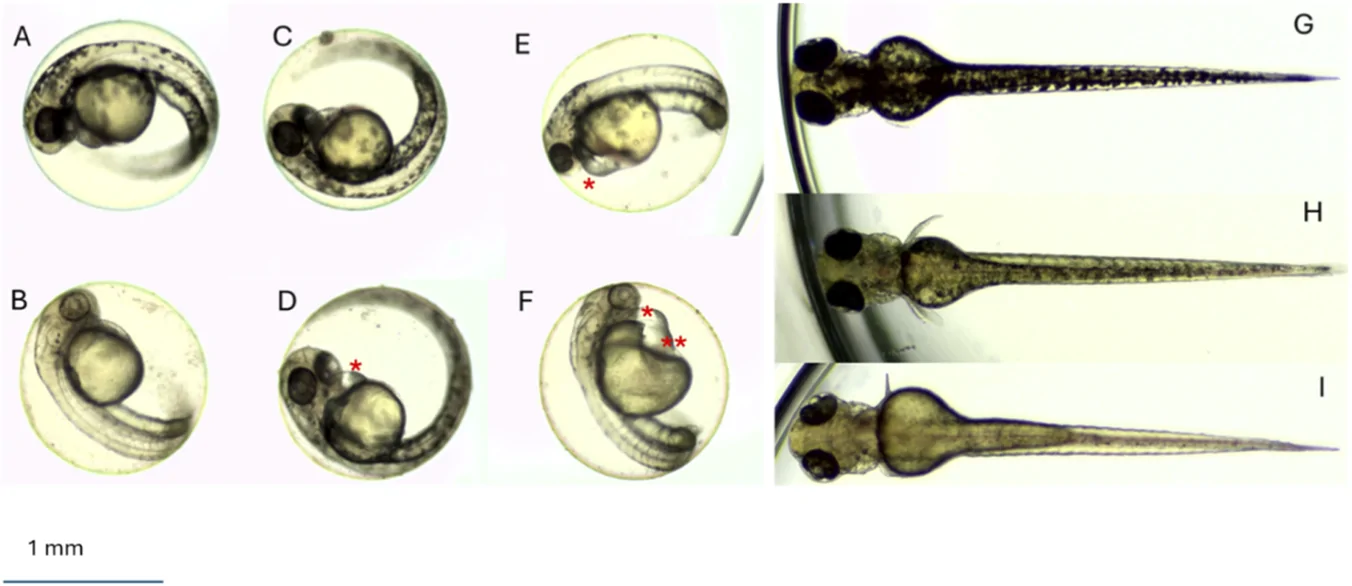
An example of morphological changes in zebrafish at 48 hpf: **(A)** control untreated, **(B)** reduced pigmentation after exposure to 2.0 mM paracetamol, **(C)** 85.5 mM ethanol, **(D)** 85.5 mM ethanol and 1.0 mM paracetamol slightly visible pericardial edema *, **(E)** 342.0 mM ethanol pericardial edema *, **(F)** 342.0 mM ethanol and 2 mM paracetamol larger and more visible pericardial edema * and visible yolk edema ** and at 72 hpf: the changes in pigmentation: **(G)** normal pigmentation in control, **(H)** reduced pigmentation after exposed to 1 mM paracetamol, **(I)** lack of pigmentation after exposure to 1 mM paracetamol and 171.0 mM ethanol.

The first noticeable swellings in zebrafish were observed at 48 hpf after exposure, primarily in the pericardial area and the yolk sac (Figure 3). When examining the effects of paracetamol alone on *D*. *rerio*, pericardial edema was seen only at a concentration of 2.0 mM of the drug in individual cases, and the difference was not statistically significant No edema was observed at lower concentrations of paracetamol. Larvae exposed to 342.0 mM ethanol exhibited pericardial edema and yolk sac edema.

The co-exposure markedly elevated the incidence of edema when using a mixture of 342.0 mM ethanol and paracetamol at concentrations of 0.5, 1.0 and 2.0 mM. During exposure to 342 mM ethanol, 71.8% of the larvae developed pericardial edema, while 15.1% showed yolk edema. Conversely, when 2.0 mM of paracetamol was combined with 342.0 mM ethanol, 100% of zebrafish larvae displayed pericardial edema, and the incidence of yolk edema rose to 64.2% (Figure 3).

### Evaluation of pigmentation changes

At 72 hpf, larvae were examined following exposure to paracetamol (0.5, 1.0 and 2.0 mM), ethanol (85.5, 171.0 and 342.0 mM), and their mixtures.

The pigmentation of the larvae was visually assessed. A three-stage scale was used to determine their coloration: P0 - loss of pigmentation, P1 - partial loss of pigmentation, P2 - normal pigmentation.

Statistical analysis: significant differences between groups were observed in the case of normal pigmentation (P2) [Kruskal–Wallis test: H = 46.62 (*p* < 0.001)], with the control group showing 100% pigmentation.

Embryos with a loss of pigmentation (P0) were also compared, and statistical differences were observed compared to the control, in which no embryos with a lack of pigmentation were observed. Test [Kruskal–Wallis test: H = 46,81 (*p* < 0.001)].

Exposure of *D*. *rerio* to ethanol alone or 0.5 mM paracetamol alone did not cause any abnormalities in their natural pigmentation. Paracetamol alone at a concentration of 1 mM slightly reduced pigmentation in zebrafish embryos, but the difference is not statistically significant. However, the reduction in pigmentation is visible if 1 mM paracetamol is administered simultaneously with 342.0 mM of ethanol (*p* < 0.01).

**FIGURE 4:**
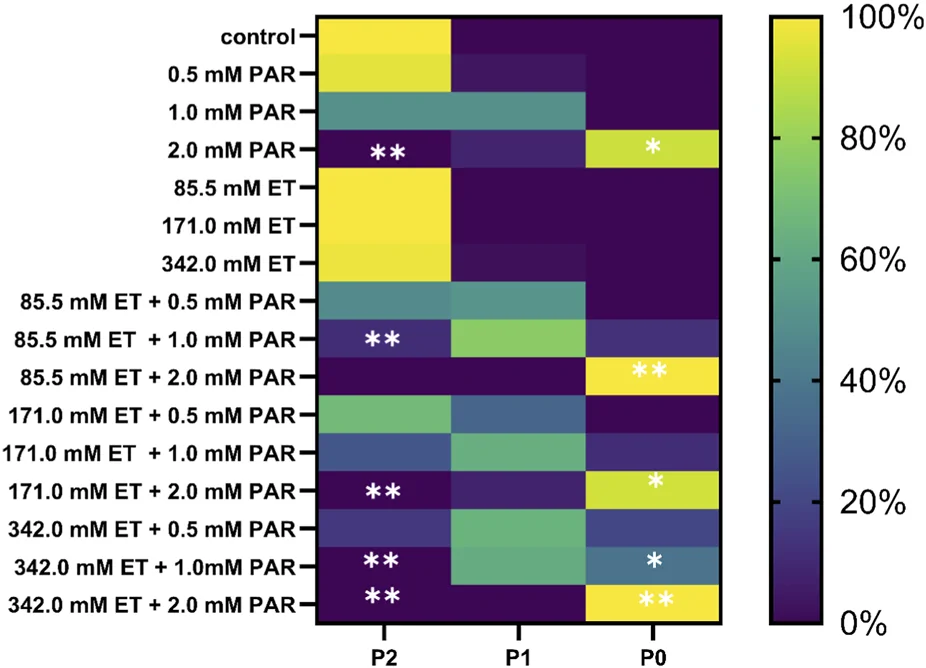
Heatmap showing the percentage of zebrafish exhibiting depigmentation phenotypes at 72 hpf after continuous exposure to paracetamol (PAR; 0.5, 1.0, and 2.0 mM), ethanol (ET; 85.5, 171.0, and 342.0 mM), and their combinations (PAR:ET ratios of 0.5:85.5, 1.0:171.0, and 2.0:342.0). The color scale represents the percentage of larvae in each category, ranging from 0% (dark purple) to 100% (yellow), as indicated by the color bar. Intermediate values are shown as a gradient from purple through blue and green to yellow. Columns indicate pigmentation categories: P0 – complete loss of pigmentation, P1 – partial loss of pigmentation, and P2 – normal pigmentation. Rows correspond to different treatment conditions. Data were analyzed using the Kruskal–Wallis test followed by Dunn's post hoc test. Asterisks denote statistically significant differences compared to the control group (*p < 0.05, **p < 0.01).

At a higher concentration of the drug alone (2.0 mM), none of the larvae displayed a normal pigmentation (p < 0.01) versus control (Figure 4).

The combination of the drug and alcohol increased the proportion of larvae with a decrease pigmentation. Higher concentrations of ethanol, while keeping the concentration of paracetamol unchanged, led to a complete disappearance of pigmentation. In the case of 2.0 mM paracetamol 91.5%, larvae exhibited partial loss of pigmentation while 100% larvae exposed to both alcohol and the drug experienced complete pigmentation loss (Figures 3, 4).

### Body length, body width and eye size in *D.rerio*

The body length, body width, and eye size of *D*. *rerio* were measured 72 hpf. An ANOVA test with *post hoc* analysis was conducted to evaluate the measurements statistically (see Table 1). Significant effects on body length [F = 4.4 (*p* < 0.001)] were observed when embryos were exposed to both paracetamol and ethanol simultaneously, particularly at higher concentrations of paracetamol combined with ethanol. However, no effects on body length were noted with paracetamol alone or ethanol alone.

**TABLE 1:** Mean length, width and eye size of larvae at 72 hpf in the control group and those exposed to 85.5; 171.0 mM ethanol, paracetamol 0.5; 1.0 and 2.0 mM and paracetamol (PAR) in combination with 85.5; 171.0 mM ethanol.

Solution	Mean length ± SD [μm]	Mean width ± SD [μm]	Mean eye size ± SD [μm]
E3 (control)	3361.56 ± 78.02	486.63 ± 15.18	278.68 ± 8.40
0.5 mM PAR	3357 ± 74.26	485.48 ± 44.23	271.76 ± 12.14
1.0 mM PAR	3321.76 ± 93.78	481.63 ± 57.53	270.48 ± 11.50
2 mM PAR	3293.83 ± 148.82	496.70 ± 17.98	240.80 ± 12.61**
85.5 mM ethanol	3363.22 ± 108.63	470.45 ± 22.83	272.63 ± 17.58
85.5 mM ethanol + 0.5 mM PAR	3297.9 0 ± 63.25	473.76 ± 22.74	268.26 ± 18.53
85.5 mM ethanol + 1.0 mM PAR	3326.23 ± 65.06	473.13 ± 20.12	263.12 ± 11.06*
85.5 mM ethanol + 2.0 mM PAR	3275.42 ± 89.34	475.45 ± 20.74	240.29 ± 15.41**
171.0 mM ethanol	3301.10 ± 84.62	479.24 ± 25.48	266.29 ± 12.37*
171.0 mM ethanol + 0.5 mM PAR	3371.95 ± 74.59	487.62 ± 48.72	264.69 ± 12.98**
171.0 mM ethanol + 1.0 mM PAR	3282.12 ± 55.44	484.14 ± 33.92	265.32 ± 14.92*
171.0 mM ethanol + 2.0 mM PAR	3265.78 ± 115.93*	519.14 ± 21.90*	242.92 ± 17.96***

Values are presented as mean ± standard deviation (SD), n = 26 Statistical significance is indicated as follows: *p < 0.05, **p < 0.01, and ***p < 0.001, with significant differences noted from the control group. Data were analyzed by one-way analysis of variance (ANOVA) followed by Tukey’s HSD test, * indicates p < 0.05, **indicates p < 0.01, ***indicates p < 0.001 versus control.

A statistically significant increase in body width was observed when the embryos were exposed to 2.0 mM paracetamol and 171.0 mM ethanol [F = 5.6 (*p* < 0.001)].

Exposure of the larvae to paracetamol solutions (0.5, 1.0, and 2.0 mM) resulted in a decrease in eye size as the concentration increased. A statistically significant reduction in eye size was observed with the 2.0 mM paracetamol solution. This trend was also seen with the combination of ethanol and paracetamol. Furthermore, statistically significant results were obtained in the group of larvae exposed to 85.5 mM ethanol combined with 1.0 mM and 2.0 mM paracetamol, as well as in all paracetamol concentrations when exposed to 171.0 mM ethanol simultaneously [F = 23.5 (p < 0.001)].

### The interactions between paracetamol and ethanol

The LC_50_ values indicate the concentration of a given substance (or their mixture) causing 50% larval mortality and were determined by the probit method in CompuSyn software, for paracetamol, it was found to be 5.57 mM, for ethanol, it was 323.6 mM, and it was 197.5 mM for the mixture of both substances used simultaneously.

**FIGURE 5:**
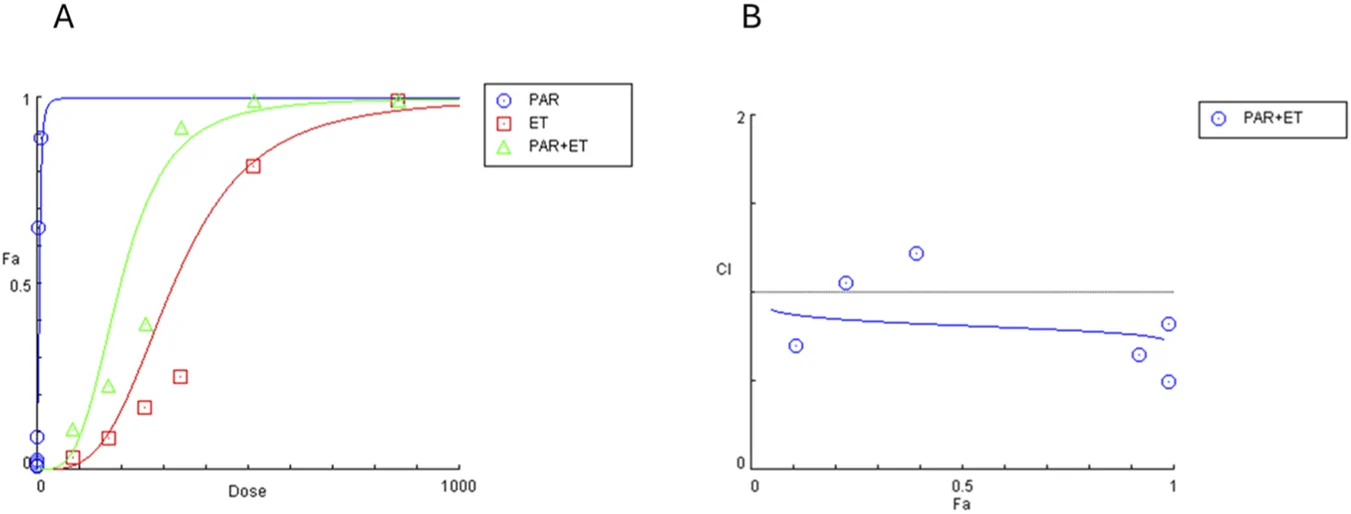
The dose-effect curve plot paracetamol and ethanol **(A)**, Fa-fraction affected; Cl-combination index of paracetamol and ethanol **(B)**; PAR–paracetamol, ET - ethanol.

The dose-effect curve illustrates the relationship between the concentration of paracetamol, ethanol, or their combination and the observed larval mortality (Figure 5A).

For the combination of paracetamol and ethanol, the combination index (Cl), which quantitatively describes the nature of the interaction between the tested substances (synergistic, additive, or antagonistic), is 0.81 with an efficacy factor Fa = 0.5 and 0.79 with an efficacy factor of Fa = 0.75. Fa parameter (fraction affected) indicates the percentage of the population affected by a given toxic effect. This suggests a synergistic effect between these substances (Figure 5B). In other words, the combined toxicity of paracetamol and ethanol is greater than the sum of their individual effects, demonstrating a synergistic interaction.

## Discussion

For many years, many *in vitro* and *in vivo* experimental models have been utilized to better understand the mechanisms and effects of the interaction between paracetamol and ethanol. The studies primarily involved human primary hepatocytes, human liver microsomes, and the Hep G2 cell line and rodents (Gonçalves et al., 2024; Moldeus et al., 1980; Neuman, 2002; Tredger et al., 1985). Across various *in vitro* and *in vivo* models multiple studies have demonstrated the significant impact of paracetamol and ethanol, used alone or in combination resulting in notable alterations in general metabolism and its regulation, including melanin synthesis alteration and pathomorphological disturbances (Adekeye and Fafure, 2022; Mladenovic et al., 2013; Neuman, 2002).

Neuman et al. (2002) conducted experiments using a model of human primary hepatocytes (NHPH) and the Hep G2 cell line. Overall, the study demonstrated that the combination of alcohol and paracetamol metabolites induces cell apoptosis *in vitro* (Neuman, 2002). Other studies that analyzed specific metabolic processes, such as glucuronidation, utilized human liver microsomes. These microsomes were induced with paracetamol, ethanol, and opioids for a specified period. An *in vitro* study found that ethanol did not inhibit paracetamol glucuronidation (Boldt et al., 2007). Additionally, another experiment involved adult male Swiss mice to investigate the effects of excessive alcohol and paracetamol consumption on oxidative stress in the liver. The mice received intravenous paracetamol and 12 h after the last administration of the drug, they were treated with ethanol. The results suggested a synergistic effect between paracetamol and ethanol, particularly regarding lipid peroxidation and liver superoxide dismutase (SOD) activity (Mladenovic et al., 2013). In subsequent analyses of this issue, healthy male rats were used as the experimental model. The simultaneous ingestion of these xenobiotics resulted in a decrease in the body weight of the rats compared to the control group. Additionally, the simultaneous administration of paracetamol and ethanol influences melatonin concentration in the retina of rats. This reduction in the neurohormone interfered with normal visual function, as melatonin acts on MT1 receptors located on photoreceptors and disrupts the regulation of circadian rhythms (Adekeye and Fafure, 2022).

In this study, a reduction in pigmentation was observed in zebrafish larvae following paracetamol exposure. Importantly, this effect appeared to be more pronounced under conditions of co-exposure with ethanol. The mechanism underlying the observed changes is unclear and requires further investigation. However, based on the available literature, several possible explanations can be identified. One is oxidative stress, which may affect melanocyte survival, migration and function (Eom et al., 2012; Pardo-Sánchez et al., 2022). Paracetamol has previously been described as a factor capable of inducing oxidative stress and leading to hypopigmentation in a zebrafish model (Dewanti et al., 2023; Rosas-Ramírez et al., 2022).

Results of previous studies have shown that paracetamol binds strongly to melanin in small amounts. As a result, the unbound drug can cause a skin rash. Wrześniok et al. (2016) conducted a study on human epidermal melanocytes, in which it was found that paracetamol at concentrations of 2.0 mM or higher reduces the activity of the enzyme tyrosinase, which is necessary for melanin biosynthesis, leading to a reduction in the amount of macromolecular pigments in melanocyte cells (Wrzesniok et al., 2016). In another study involving zebrafish exposed to concentrations of 2.5 mM, 4.9 mM, and 6.9 mM of paracetamol, pigmentation loss was observed at 96 hpf (David and Pancharatna, 2009).

Additionally, ethanol affects neural crest cells, from which melanocytes originate, disrupting their development, migration or differentiation. Changes in zebrafish pigmentation were observed after exposure to 171 mM ethanol alone at 8 days post-fertilization (dpf) (Zavareh et al., 2022) but these changes were not visible in our study at 72 hpf.

The present study revealed that pigmentation in zebrafish larvae was reduced after exposure to paracetamol, and co-exposure to ethanol significantly intensified this effect, even at lower paracetamol concentrations when administered simultaneously with ethanol.

Previous studies on the effects of paracetamol on the embryonic development of zebrafish have shown a variety of morphological and physiological abnormalities, the occurrence of which depended on the drug concentration used (Cedron et al., 2020; David and Pancharatna, 2009; Dewanti et al., 2023). The results of the present study, which examined the effects of paracetamol alone on zebrafish embryos, aligned with previous findings that reported morphological changes and pigmentation disorders.

In the present study, the LC_50_ for paracetamol is comparable to the study by Cedron et al. in which the LC_50_ value was 6.668 mM at 96 h of exposure to acetaminophen (Cedron et al., 2020). Similarly, Ali, van Mil, and Richardson (2011) found an LC_50_ of 3.54 mM within the same time frame (Ali et al., 2011) and Calvarho et al. (2024) 5.15 mM (de Carvalho et al., 2024). The LC_50_ values obtained in this study were within the range of 3.5–6.7 mM reported by previous studies, validating the reliability of our model.

Numerous studies have examined the effects of ethanol on the development of *D. rerio*. These studies describe various morphological, behavioral, and molecular changes, as well as the immediate and long-term consequences of exposure to ethanol. Exposure of embryos to ethanol resulted in pericardial edema, yolk sac edema, and tail curvature (Malkowska et al., 2024). Furthermore, larvae exposed to 1.0% ethanol exhibited a statistically significant reduction in body length compared to the control group (Pinheiro-da-Silva & Luchiari, 2021). However, Ramlan et al. (2017) reported a significant decrease in length only at higher concentrations of 1.5% and 2.0% (Ramlan et al., 2017).

A review of studies examining embryo survival at different ethanol concentrations revealed varied results. For example, Fu et al. conducted a survey where embryos were exposed to ethanol for short periods (from 6 to 24 h post-fertilization). They recorded 0%, 17%, and 100% mortality rates for concentrations of 0.5% (85.5 mM), 1.5% (256.5 mM), and 3.0% (513 mM) respectively at 120 hpf (Fu et al., 2021). Our study observed a 25% mortality rate after exposure to 2% (342.0 mM) ethanol. The LC_50_ value for ethanol at 72 hpf was determined to be 323.6 mM. This result aligns with findings from Busquet et al. (2014), who reported LC_50_ values of 285.8 mM at 48 hpf and 259.1 mM at 96 hpf (Busquet et al., 2014) or 240 mM specified by Lammer et al. (2009) at 48 hpf in zebrafish (Lammer et al., 2009).

Understanding the mechanisms of interactions between drugs and alcohol is a significant challenge in the field of medicine. These interactions can be difficult to predict, hindering effective healthcare and compromising patient safety. Several decades ago, studies began investigating the interaction between paracetamol and ethanol, but further research is needed to clarify many unresolved questions regarding this interaction (Prescott, 2000; Tanaka et al., 2000). Additionally, there have been no studies conducted on the interaction between ethanol and paracetamol using the zebrafish model.

The current study investigated the effects of paracetamol and ethanol on the normal development of zebrafish embryos. We found an increase in both lethal and sublethal lesions when paracetamol and ethanol are administered simultaneously compared to the effects of the two substances administered alone. Furthermore, higher concentrations of the test substances intensified these changes. This relationship was particularly evident when comparing the lethal effects of 342.0 mM ethanol alone and with exposure combined with paracetamol at 1.0 and 2.0 mM (Figure 1). Notably, in terms of eye size measurements, we observed that ethanol exacerbated the mean reduction of eyes in lower concentrations of paracetamol (0.5 mM and 1 mM), in which, when administered alone, the reduction of eyes in zebrafish is not statistically significant (Table 1).

Potential biological mechanisms that may explain the toxic effects of paracetamol, ethanol, and their combined effects are: induction of cytochrome P450 enzymes (particularly CYP2E1), depletion of glutathione (GSH) -a key element in NAPQI detoxification, observed in condition of co-exposure to paracetamol and ethanol, disturbances in mitochondrial function and cellular energy homeostasis, leading to the activation of apoptotic and necrotic pathways and increased oxidative stress and inflammatory response (Hedgpeth et al., 2019; Kumar et al., 2022).

The Chou-Talalay method was used to assess the synergistic effects of combining two xenobiotics: paracetamol and ethanol. The determined LC_50_ value for the paracetamol-ethanol combination was 197.5 mM, indicating that this mixture is more toxic than paracetamol alone. Numerous studies have explored this interaction across various experimental models, primarily focusing on hepatotoxicity (Boldt et al., 2007; Mladenovic et al., 2013). This study is novel as it highlights the synergistic effect of ethanol and paracetamol using the zebrafish model.

This study demonstrates that co-exposure to paracetamol and ethanol markedly enhances developmental toxicity in zebrafish embryos, as evidenced by increased mortality, more severe morphological abnormalities, and a pronounced reduction in pigmentation compared to single-compound exposure.

Analysis using the Chou–Talalay method confirmed a synergistic interaction between these compounds, indicating that ethanol potentiates paracetamol toxicity even at lower concentrations.

These findings underscore the importance of considering combined exposure to commonly used substances such as paracetamol and ethanol, particularly in the context of early development. Further research are needed to elucidate the underlying molecular mechanisms, with particular emphasis on oxidative stress and metabolic pathways.

## Data Availability

The raw data supporting the conclusions of this article will be made available by the authors, without undue reservation.
